# Influence of Drying Temperature on Quality Characteristics and Drying Kinetics of *Siraitia grosvenorii* Fruit

**DOI:** 10.3390/foods15020335

**Published:** 2026-01-16

**Authors:** Li Li, Ting Gan, Lihong Xie, Ping Yi, Yuhan Long, Min Huang, Dan Luo, Lan Zhang, Fenglai Lu, Jian Sun, Dianpeng Li

**Affiliations:** 1 School of Chemistry and Chemical Engineering, Guangxi University, Nanning 530004, China; lili@gxaas.net (L.L.); danluo@gxaas.net (D.L.); 2 Guangxi Academy of Agricultural Sciences, Nanning 530007, China; ganting@gxaas.net (T.G.); lihong@gxaas.net (L.X.); pingyi@gxaas.net (P.Y.); yuhan__l1998@163.com (Y.L.); hmin@gxaas.net (M.H.); zhanglan2023@gxaas.net (L.Z.); 3 Key Laboratory of Plant Functional Phytochemicals and Sustainable Utilization, Guangxi Institute of Botany, Guangxi Zhuang Autonomous Region and Chinese Academy of Sciences, Guilin 541006, China; lufenglai@gxib.cn; 4 Guangxi Academy of Sciences, Nanning 530007, China

**Keywords:** *Siraitia grosvenorii*, hot-air drying, drying kinetics, antioxidant activity, mogrosides

## Abstract

*Siraitia grosvenorii* fruit, a traditional medicinal and edible plant, undergoes significant alterations in quality and bioactive composition during the dehydration process. This study investigated the effects of hot-air drying at various temperatures on the physicochemical properties, antioxidant activity, and drying kinetics of *S. grosvenorii* fruit. The drying process was terminated when fruit moisture content reached 15%, with corresponding drying durations of 420, 225, 144, 96, and 51 h at 40 °C, 50 °C, 60 °C, 70 °C and 80 °C, respectively. Among the ten mathematical models evaluated, the Midilli–Kucuk model provided the most accurate description of the drying kinetics of *S. grosvenorii* fruit. Quality analysis revealed that drying reduced the sugar/acid ratio, contents of mogrosides and ascorbic acid, while increasing total phenolic and flavonoid levels. Microstructural analysis revealed that higher temperatures increased drying rates by expanding the porosity of the pulp. Based on the retention of bioactive components and antioxidant capacity, 70 °C was identified as the optimal drying temperature. Overall, these findings suggest that oven-drying optimizes drying efficiency and ensures the retention of essential bioactive constituents in *S. grosvenorii*.

## 1. Introduction

*Siraitia grosvenorii*, commonly known as monk fruit or Luo Han Guo, is a perennial vine belonging to the *Cucurbitaceae* family [1]. Indigenous to southern China, this plant holds significant value in both traditional Chinese medicine and the food industry, given its dual medicinal and edible properties [2]. Previous studies have identified over 100 active ingredients in *S. grosvenorii* fruit, including cucurbitane-type triterpene glycosides (e.g., mogrosides), flavonoids, polysaccharides, proteins, amino acids, and essential oils, which have been shown to benefit human health [3,4]. Recent pharmacological studies have demonstrated that *S. grosvenorii* exhibits a broad spectrum of bioactive properties, including antioxidant, anticancer, anti-asthmatic, anti-inflammatory, antiglycation, and hepatoprotective effects [2,5]. Mogrosides constitute the primary chemical constituents of the *S. grosvenorii* fruit, and have been reported to be calorie-free, and are more than 300-fold sweeter than sucrose. Mogrosides have been approved as a natural sweetener in health foods for individuals with obesity and diabetes [6]. In addition, phenols and flavonoids are significant bioactive compounds present in *S. grosvenorii* fruit, exhibiting antibacterial and antioxidant properties [3].

Fresh *S. grosvenorii* fruit is highly susceptible to postharvest decay and enzymatic browning owing to its high moisture content. Accordingly, drying is a crucial processing step for inhibiting microbial proliferation and extending storage stability. In China, commercial *S. grosvenorii* fruits are primarily distributed in dried form, with a standard final moisture content of 15%, which was the recommended moisture content for safe storage of dried *S. grosvenorii* fruit [7]. Extensive studies have confirmed that dehydration substantially alters the native phytochemical profile, thereby affecting both bioactive concentrations and functional properties [8]. Research has consistently demonstrated that increasing drying temperatures negatively impacts key quality attributes. For instance, the concentrations of both mogrosides and monosaccharides exhibit a declining trend as temperatures increase [9]. Fang et al. reported that hot-air drying significantly reduced the total mogroside content while facilitating the conversion of mogroside V into lower-molecular-weight glycosides [10]. Similarly, a significant decrease in mogroside V, vitamin C, protein, fructose, and glucose in dried *S. grosvenorii* fruit compared with their fresh fruit counterparts was reported by Hu et al. [11]. In contrast, dried fruits exhibit significant increases in phenolics, flavones, and flavonols compared to fresh samples [8]. These findings collectively indicate that hot-air drying significantly affects the composition of *S. grosvenorii* fruit. However, the effects of different drying temperatures on the bioactive compounds of *S. grosvenorii* fruits warrant further investigation.

Drying is a crucial processing step for *S. grosvenorii* fruit, significantly affecting final product quality. Traditional drying methods primarily rely on high-temperature baking (above 90 °C), which often yields undesirable outcomes, including scorching, discoloration, and nutrient degradation resulting from excessive thermal exposure. Among contemporary drying technologies, hot-air drying has emerged as one of the most prevalent methods due to its technical simplicity, straightforward operation, and economic benefits [12]. In recent years, numerous mathematical models have been developed to characterize drying processes [13]. These models describe the process of water removal from porous media through evaporation until moisture equilibrium is attained [14]. Mathematical modeling has proven particularly effective for simulating drying kinetics and elucidating moisture transfer mechanisms, offering valuable insights for process control. Nevertheless, few studies have specifically investigated the mathematical modeling of hot-air drying kinetics for *S. grosvenorii* fruit.

This study aimed to evaluate the influence of hot-air drying temperatures (40–80 °C) on the retention of bioactive compounds in *S. grosvenorii* fruit, with emphasis on physicochemical and microstructural characteristics and antioxidant capacity. Furthermore, to describe the observed changes in water content during the drying tests, the moisture ratios were fitted using theoretical models and empirical models found in the literature. The drying models used in this research can be very important tools to estimate the drying behavior under different drying conditions and to optimize the drying process. These results provide valuable guidance for optimizing drying conditions to effectively preserve the bioactive components of *S. grosvenorii* fruit.

## 2. Materials and Methods

### 2.1. Sample Collection and Drying of Siraitia grosvenorii Fruits

Fresh *S. grosvenorii* fruits at commercial maturity (after 80 d of pollination) were harvested from Yongfu County, Guangxi Province, China (latitude 109.98° N, longitude 24.98° E), and immediately transported to a laboratory at Guangxi Academy of Agricultural Sciences. They were carefully selected for uniformity in size (transverse diameter 55 mm–58 mm), consistent yellowish coloration (indicating full maturity), and the absence of physical damage or disease symptoms.

Thirty fruits were sampled for baseline characterization. The remaining 300 fruits were randomly divided into five experimental groups corresponding to different hot-air drying temperatures (40 °C, 50 °C, 60 °C, 70 °C, and 80 °C). Fruits were arranged in a single layer on perforated stainless-steel trays and processed in an electric convection drying oven (WGL-230B, Tianjin Test Instrument Co., Ltd., Tianjin, China) with a constant air velocity of 2 m/s. In all experimental runs, dehydration continued until the moisture content reached 15% (wet basis), which was the recommended moisture content for safe storage of commercial dried *S. grosvenorii* products [11]. All drying trials were conducted in triplicate (*n* = 3) using a completely randomized design.

### 2.2. Drying Characteristics

#### 2.2.1. Moisture Ratio

To determine moisture content, three fruits per treatment were sampled and individually homogenized with a mechanical mill (MM 400, Retsch GmbH, Haan, Germany). The dry weight (DW) of the fruits was obtained after drying the fruits in an oven (WGL-230B, Taisite Instrument Co., Ltd., Tianjin, China) at 105 °C until the mass of the sample no longer changed. The moisture content of *S. grosvenorii* fruits throughout the drying process was calculated as follows [15]:

Moisture Content (g/g) = (*m_t_* − *m_d_*)/*m_d_* (1)

*m_t_*: the sample weight at time *t* (g); *m_d_*: the absolute dry weight (g).

The drying rate (DR) of *S. grosvenorii* fruits was determined as follows [14]:

Drying Rate = (*M*_*t*+__Δ*t*_ − *M_t_*)/Δ*t* (2)

*M_t_*: the moisture content at time *t* (g water/g DW); *M*_*t*+__Δ*t*_: the moisture content at time *t* + Δ*t* (g water/g DW); Δ*t*: the time interval between measurements (hours).

To determine the drying kinetics of *S. grosvenorii* fruits, moisture content data were analyzed to construct drying curves. These curves illustrate the relationship between moisture ratio (*MR*), the independent variable, and time, the dependent variable. The moisture content values obtained at various drying temperatures were normalized using the following dimensionless moisture ratio equation [14]:

Moisture Ratio (%) = (*M_t_* − *M_e_*)/(*M_i_* − *M_e_*) (3)

*M_t_*: the monitoring time point moisture content (g water/g DW); *M_i_*: the initial moisture content (g water/g DW); *M_e_*: the equilibrium moisture content (g water/g DW). In cases where *M_e_* is considerably smaller than *M_i_*, it can be considered negligible.

#### 2.2.2. Modeling of Drying Kinetics

The drying kinetics were characterized by evaluating the moisture ratio as a function of time. To identify the optimal model describing the drying behavior of *S. grosvenorii* fruits, ten widely used thin-layer drying models were evaluated (Table 1), following the methodology established by Ertekin and Firat [16]. These models were selected based on their demonstrated efficacy in representing the drying kinetics across various agricultural commodities.

Model fitting performance was quantitatively assessed using the coefficient of correlation (*R*^2^), values of chi-square (*χ*^2^), and root-mean-square error (RMSE). The model parameters and *R*^2^ values were calculated using nonlinear regression in Origin Pro 2024 (OriginLab Inc., Northampton, MA, USA). A higher *R*^2^ value, coupled with lower *χ*^2^ and RMSE values, indicated a superior fit of the established model. These parameters were calculated according to the following equations [17]:

(4) $$R^{2}=1-\left[\sum_{i-1}^{N}{\left({MR}_{exp,i}-{MR}_{pre,i}\right)}^{2}/\sum_{i-1}^{N}{\left({MR}_{exp,i}-\bar{{MR}_{exp,i}}\right)}^{2}\right]$$

(5) $$\chi^{2}=\sum_{i=1}^{N}{\left({MR}_{exp,i}-{MR}_{pre,i}\right)}^{2}/\left(N-n\right)$$

(6) $$RMSE={\left[1/n\sum_{i=1}^{N}{{(MR}_{exp,i}-{MR}_{pre,i})}^{2}\right]}^{1/2}$$

*N*: the total number of observations; *n*: the number of constants; *MR_exp__,i_*: the experimental moisture ratios; *MR_pre__,i_*: the predicted moisture ratios; $\bar{{MR}_{exp,i}}$: the mean of the experimental dimensionless moisture ratio values.

### 2.3. Quality Parameters

#### 2.3.1. Color Properties

The color profiles of *S. grosvenorii* fruit were quantitatively analyzed using a portable colorimeter (CS-412, CHNSpec Technology Co., Ltd., Hangzhou, China). The instrument was configured with a D65 light source and an 11 mm measurement aperture. Color values were expressed by L* (brightness), a* (red or green), and b* (yellow or blue) [18]. Measurements were conducted in triplicate, with results expressed as mean values. The distance between dried samples and fresh fruit can be expressed as the total color difference (Δ*E*), which was calculated using the following equation:

(7) $$∆E=\sqrt{{\left(L^{*}-L_{0}^{*}\right)}^{2}+{\left(a^{*}-a_{0}^{*}\right)}^{2}+{\left(b^{*}-b_{0}^{*}\right)}^{2}}$$

#### 2.3.2. Maturity Index

Maturity index is expressed as the sugar/acid ratio, calculated by dividing total soluble solids (TSS) by the titratable acidity (TA) of the given sample [19]. TSS content was determined according to the method described by Zhang et al. [20] with minor modifications. TSS content was determined by a digital refractometer (PAL-1, ATAGO, Tokyo, Japan) and was presented in °Brix. TA was conducted using the method described by Al-Dairi et al. [19], and the TA content was expressed as a percentage of malic acid.

#### 2.3.3. Microstructural Analysis

The microstructural features of dried *S. grosvenorii* pulp were characterized using scanning electron microscopy (SEM) according to the modified method of Ju et al. [21]. *S. grosvenorii* pulp samples were precisely sectioned into uniform 1.0 mm cubic specimens. Prior to imaging, all samples were sputter-coated with a 10 nm gold layer using an MC1000 sputter coater (Hitachi, Tokyo, Japan) for 30 s to enhance surface conductivity. SEM imaging was performed using an SU-8100 field-emission microscope (Hitachi) operated at an acceleration voltage of 3 kV under high-vacuum conditions (10^−3^ Pa), with working distance maintained at 8 mm for optimal resolution. All samples were imaged at room temperature at appropriate magnifications to capture the detailed microstructure of the specimens.

#### 2.3.4. Determination of Mogroside Content

Mogroside content was analyzed using ultra-performance liquid chromatography (UPLC) following a modified method by Pei et al. [22]. Sample powders (0.1 g) of *S. grosvenorii* were subjected to ultrasonic extraction with 10 mL of 20% (*v*/*v*) methanol for 30 min (120 W, 40 kHz), then filtered through a 0.45 μm membrane to obtain the test solution.

UPLC analysis was performed on an Acquity UPLC H-Class system (Waters, Milford, MA, USA) equipped with an Acquity UPLC HSS T_3_ analytical column (100 mm × 2.1 mm × 1.8 μm, Waters). Chromatographic separation was achieved using a binary gradient elution system consisting of water (A) and acetonitrile (B) with the following gradient elution program: 0–2 min (A/B = 80/20), 2–10 min (80%→73% A), 10–11 min (A/B = 73/27), 11–16 min (73%→10% A), 16–18 min (10%→80% A) and 18–22 min (A/B = 80/20). Flow rate was maintained at 0.3 mL/min, injection volume was 5.0 µL, and column temperature was 35 °C. Detector wavelength was set to 203 nm. The content of mogrosides in fruit dry weight (DW) was expressed as g/100 g DW.

#### 2.3.5. Determination of Total Phenolic, Total Flavonoid, and Ascorbic Acid Contents

Total phenolic content was determined according to the method described by Hamid et al. [23] with minor modifications. Anhydrous methanol with 1% hydrochloric acid was used to extract phenolic compounds from the powder of *S. grosvenorii* fruits. Total phenolic content was presented as mg of gallic acid equivalent (GAE)/g of dried extract. Total flavonoid content was determined according to the method described by Liu et al. [24], with slight modifications. Total flavonoid concentration was determined from a seven-point rutin calibration curve and expressed as mg of rutin equivalents per 100 g of dry weight (mg RE/100 g DW). Ascorbic acid content was evaluated according to the method described by Huang et al. [25] with slight modifications. A 0.3 g sample of *S. grosvenorii* fruit powder was mixed with 10 mL of a 50 g/L trichloroacetic acid solution. The mixture was homogenized, incubated for 10 min, and subsequently filtered; the resultant supernatant was collected as the sample extract. Subsequently, 0.5 mL of this extract was pipetted into a tube containing 1.5 mL of 50 g/L trichloroacetic acid solution and thoroughly mixed. This mixture was then allowed to react at 30 °C for 1 h. An ethanolic solution of ascorbic acid was utilized to establish a standard calibration curve. Absorbance measurements for both samples and standards were performed spectrophotometrically at 534 nm. Results are expressed as mg per 100 g on a dry weight basis.

#### 2.3.6. Determination of Antioxidant Capacity

For antioxidant capacity assessment, sample preparation was performed according to the modified method of Bhat et al. [26]. A total of 0.3 g of powdered sample was extracted by soaking in 1.0 mL of absolute methanol solution for 30 min at room temperature. The mixture was then centrifuged at 12,000× *g* for 20 min at 25 °C, and the resulting supernatant was collected as the test extract.

Total antioxidant capacity (T-AOC) was evaluated using standardized commercial assay kits (Solarbio Technologies Co., Ltd., Beijing, China) according to the manufacturer’s instructions. The T-AOC level was expressed as μmol/g DW.

2,2-Diphenyl-1-picrylhydrazyl (DPPH) radical scavenging capacity was evaluated according to the method described by Wang et al. [27], with modifications. Briefly, a 30 μL sample extract was mixed with 170 μL of DPPH-methanol solution (0.1 mM). Parallel control reactions, in which pure methanol replaced the sample extract, were performed to baseline absorbance. Following a 30 min incubation in the dark, the absorbance at 517 nm was measured to quantify the remaining DPPH concentration.

The capacity to scavenge hydroxyl radicals (•OH) was evaluated following the method described by Wang et al. [27], with slight modifications. Briefly, 0.5 mL of the sample solution was mixed with 1 mL of 9 mM FeSO_4_ and 1 mL of 8.8 mM H_2_O_2_, then incubated at 37 °C for 10 min. Subsequently, 1 mL of 9 mM salicylic acid was added, and the reaction mixture was allowed to proceed for 30 min. Absorbance was measured at 510 nm, and results were calculated as a percentage of inhibition.

Reducing power (RP) was determined according to the protocol described by Alkaltham et al. [28], with minor adjustments. A reaction mixture containing 50 μL of sample extract, 250 μL of potassium ferricyanide (10 g/L), and 250 μL of phosphate buffer (0.2 M, pH 6.6) was incubated at 50 °C for 20 min. After cooling, 80 μL of distilled water and 20 μL of ferric chloride solution (g/L) were added. Absorbance was measured at 700 nm against a blank, which contained all reagents except the sample extract.

### 2.4. Statistical Analysis

Data processing was performed using OriginPro 2024 (OriginLab Inc., Northampton, MA, USA). Statistical analyses, including one-way analysis of variance (ANOVA) and Duncan’s multiple range test, were performed using SPSS 26.0 (IBM, Inc., Armonk, NY, USA). Pearson correlation analysis was applied to evaluate relationships between compound contents and antioxidant capacity. Results were expressed as mean ± standard error (SE) from triplicate experiments (*n* = 3). Statistical significance was defined as *p* < 0.05, with *p* < 0.01 indicating highly significant differences.

## 3. Results and Discussion

### 3.1. Moisture and Drying Kinetics

**Figure 1:**
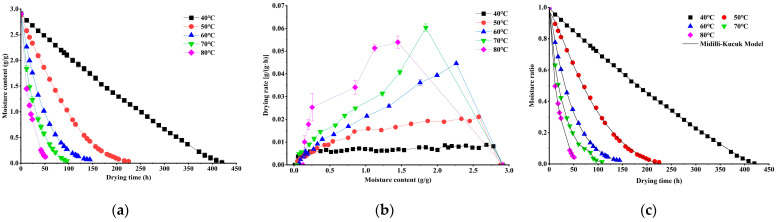
Moisture content (**a**), drying rate (**b**), and moisture ratio (**c**) of *S. grosvenorii* fruit during the drying process at different temperatures.

The fresh *S. grosvenorii* fruit exhibited an initial moisture content of 74.5 ± 0.8%, consistent with the literature [11]. As shown in Figure 1a, the moisture content decreased progressively during the drying process. The time required to achieve the target moisture content (15.0%) varied significantly with temperature, specifically 420, 225, 144, 96, and 51 h at 40 °C, 50 °C, 60 °C, 70 °C, and 80 °C, respectively. Notably, increasing the drying temperature from 40 °C to 80 °C reduced processing time by 87.5%, highlighting the substantial impact of thermal energy on drying efficiency. This acceleration could be attributed to the higher heat transfer rate at elevated temperatures, which enhanced moisture removal [14]. Similar drying kinetics have been reported for walnut [17] and *Pleurotus eryngii* [29], demonstrating the broad applicability of this thermal drying phenomenon across agricultural products.

The drying rate of *S. grosvenorii* fruit exhibited a characteristic two-phase pattern, with an initial rapid increase followed by a progressive decline as moisture content decreased (Figure 1b), which indicated that the *S. grosvenorii* fruit drying process is controlled by the internal mass transfer rate and the transfer mechanism is diffusion [17]. This drying rate curve is a typical drying behavior for food materials with porous structures or cellular structures, i.e., walnut [17] and *Pleurotus eryngii* [29]. This behavior could be attributed to the strong vapor pressure gradient between the fruit surface and the drying environment during the early stages, which facilitated efficient water removal. As drying progressed, the increasing difficulty of internal moisture migrating to the surface resulted in decreased drying rates [15].

The moisture content data of *S. grosvenorii* fruit obtained at different drying temperatures were converted into *MR* and subsequently fitted to ten thin-layer drying models, including theoretical models and empirical models (Table 1). The optimal model was selected based on the highest *R*^2^ and the lowest *χ*^2^ and RMSE values. The statistical computing results were shown in Table S1. The Midilli–Kucuk model was identified as the optimal model for the dehydration kinetics of *S. grosvenorii* fruit, based on its highest *R*^2^ value (>0.999) and lowest *χ*^2^ (<1 × 10^−4^) and RMSE (<1 × 10^−2^) values (Table 2). The model validation study revealed excellent consistency between experimental *MR* values and model predictions (Figure 1c), with a Pearson correlation coefficient of 0.9995. These results robustly validated the model’s accuracy in characterizing *S. grosvenorii* drying behavior. The Midilli–Kucuk model has also been successfully applied to describe drying kinetics in other plants, including strawberry [30], papaya [31], and turmeric [18]. As shown in Table 2, the parameter *k* in the Midilli–Kucuk model exhibited a temperature-related increase. According to Ertekin and Firat [16], parameter *k* represents the drying speed constant, crucial for effective diffusivity in the drying process during the declining period, influencing liquid diffusion, and controlling the overall process. The n parameter obtained values ranging from 0.78 to 1.25, and this value lowered with an increase in drying temperature. Parameters a and b did not present any wide variations with changes in drying temperatures.

### 3.2. Physico-Chemical Parameters

**Figure 2:**
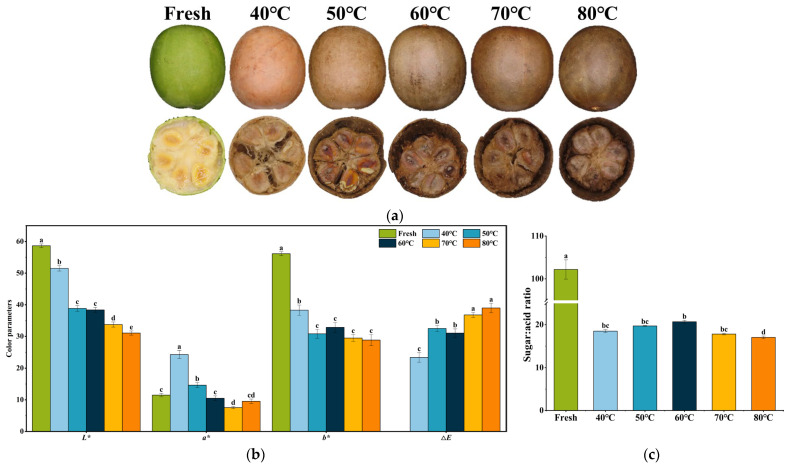
Color parameters and maturity index of *S. grosvenorii* fruit subjected to different drying temperatures. (**a**) appearance of *S. grosvenorii* fruit, (**b**) color parameters L*, a*, b*, and Δ*E*, (**c**) the sugar/acid ratio (maturity index). Different letters indicate statistically significant differences among drying temperature treatment groups (*p* < 0.05).

The drying process induced significant color changes in *S. grosvenorii* peel, transitioning from yellow to brown due to water loss and thermal effects. As shown in Figure 2a, the distinct color variations observed across different drying temperatures were strongly correlated with both thermal intensity and process duration. These observations aligned with previous reports of fruit darkening during *S. grosvenorii* dehydration [11]. Upon reaching the target moisture content of 15%, a clear temperature-dependent color gradient was observed: samples dried at 80 °C developed the darkest peel coloration, while those dried at 40 °C maintained the lightest appearance. Quantitative color analysis (Figure 2b) revealed significant temperature effects on all measured parameters (L*, a*, b* and Δ*E*). Lightness (L*) showed an inverse relationship with temperature, while Δ*E* increased proportionally with drying temperature. A higher Δ*E* value signifies more pronounced differences in color perception between dried samples and fresh fruit. It is generally accepted that Δ*E* = 1 represents the minimum perceptible color difference discernible by the average observer. The minimal Δ*E* value (25.44) was documented at 40 °C, indicating the least color deviation from fresh samples. In contrast, the maximum Δ*E* value (38.47) was observed at 80 °C, reflecting severe darkening. The observed browning phenomenon in *S. grosvenorii* fruits during high-temperature drying could be attributed to enhanced non-enzymatic reactions, particularly Maillard reactions and sugar caramelization. These results were consistent with established thermal degradation models for plant tissues such as garlic [32], kiwifruit [33], and banana peel [34], confirming that temperature is the primary determinant of pigment transformation kinetics. Figure 2c showed that the sugar/acid ratio significantly decreased after drying. However, there was no significant change in the sugar/acid ratio of fruits dried at 40–70 °C. The sugar/acid ratio at 80 °C was lowest, because the high-temperature drying might accelerate sugar inversion and caramelization to reduce the total sugar content [19].

### 3.3. Microstructural Characterization

**Figure 3:**
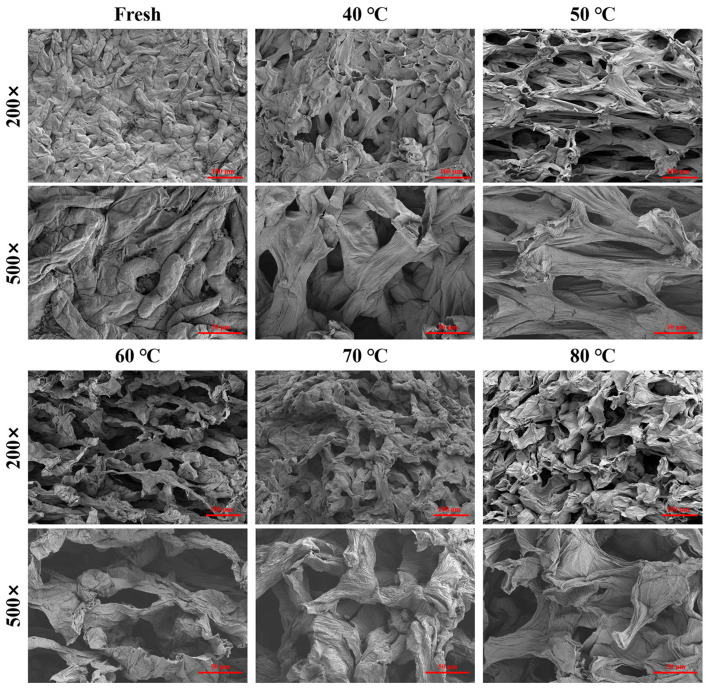
Internal structure by scanning electron microscopy technique of *S. grosvenorii* pulp dried at different hot air temperatures. Magnification: 200× and 500×.

Microstructural characterization was performed on both fresh and dried *S. grosvenorii* pulp samples, with the latter subjected to various drying temperatures (Figure 3). Fresh pulp exhibited a typical fleshy structure with intact vascular bundles and well-organized cellular architecture. The microstructural analysis of *S. grosvenorii* dried at different temperatures revealed the development of distinct porous networks showing honeycomb morphology, resulting from water loss during dehydration [21]. During the drying process, intracellular water transport leads to cell shrinkage, pore formation, and cell collapse. Ultimately, the overall flesh tissue undergoes deformation due to water migration from the cell’s interior [13]. In the present study, among the dried samples, the pulp dehydrated at 40 °C exhibited a relatively uniform pore distribution, whereas the 50 °C treatment resulted in enlarged pore formation. In fruits dried at 80 °C, cells underwent loss of structural integrity, with no discernible cellular organization remaining. These morphological changes correlated with accelerated drying rates and internal stresses arising from rapid moisture evaporation, which contributed to expanded pore size and damaged cellular integrity [35].

### 3.4. Bioactive Compound Composition

**Figure 4:**
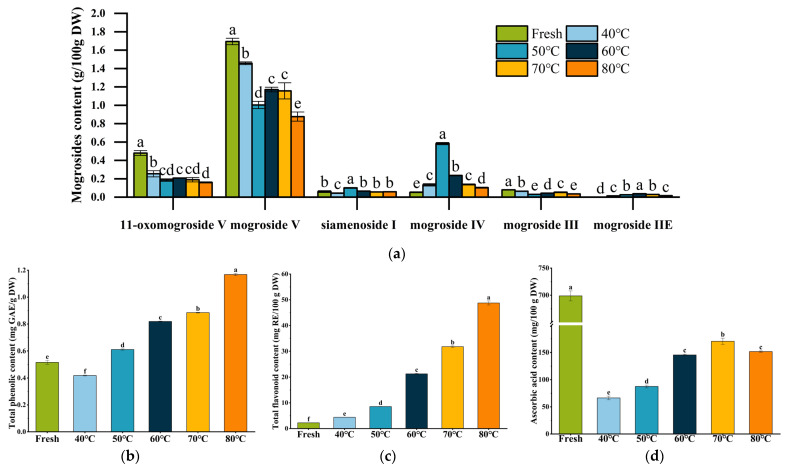
Functional constituent contents of *S. grosvenorii* before and after hot-air drying. (**a**) mogroside content; (**b**) total phenolic content; (**c**) total flavonoid content; (**d**) ascorbic acid content. Different letters in the above columns indicate significant differences among drying temperature treatment groups (*p* < 0.05).

The major bioactive sweeteners in *S. grosvenorii* fruits, including 11-oxomogroside V (11-O-MV), siamenoside I (SIA), mogrosides V (MV) and IV (MIV), are important indicators of fruit quality [36]. As illustrated in Figure 4a, the retention of these mogrosides was significantly influenced by drying temperature (*p* < 0.05). Notably, hot-air drying consistently reduced the content of mogrosides compared to fresh fruit. These results were consistent with those of Hong et al., who found that low-temperature drying yields superior preservation of 11-O-MV and MV levels than high-temperature treatments [9]. Further analysis revealed temperature-dependent variations in mogroside accumulation: 11-O-MV and MV contents were maximized at 40 °C, SIA and MIV accumulation peaked at 50 °C, while MIIE content reached its maximum at 60 °C. Although mogrosides are generally considered thermally stable [37], their degradation during drying may be attributed to the thermal inactivation of mogroside biosynthetic enzymes, including squalene epoxidase, epoxide hydrolase, cucurbitadienol synthase, and UDP-glucosyltransferases [9,38,39]. It has been reported that squalene epoxidase exhibits marginal thermostability, with a melting temperature below 42 °C [40], suggesting rapid denaturation at elevated temperatures. The optimal temperatures for key enzymes involved in MV biosynthesis were determined as follows: epoxide hydrolase exhibited maximum activity at 40 °C, while the UDP-glucosyltransferases UGTMG1, UGTMS1-M7, and UGTMS2 showed peak activity at 50 °C, 45 °C, and 45 °C, respectively [41,42]. These findings suggest that at elevated temperatures, enzyme inhibition likely disrupts mogroside biosynthesis, resulting in reduced content. In this study, even at 40 °C, the contents of 11-O-MV and MV decreased significantly compared with fresh *S. grosvenorii* samples. These findings aligned with previous reports showing that the rate of MV degradation during drying increases with elevated temperatures [11]. The results indicated that the degradation of MV in the process of drying would produce a variety of secondary glycosides, and the content of various saponins would reach a dynamic equilibrium, thus maintaining the basic stability of the total mogroside content.

The differences in total phenolic and flavonoid contents of *S. grosvenorii* fruit under different hot air-drying conditions are shown in Figure 4b,c. Drying treatment exerted a significant impact on phenolic and flavonoid contents of *S. grosvenorii* fruit. In this study, the dried *S. grosvenorii* fruits exhibited higher phenolic and flavonoid concentrations compared to fresh fruits, consistent with results reported by Hu et al. [11]. Furthermore, total phenolic and flavonoid contents exhibited a positive correlation with drying temperature. The maximum total phenolic (1.17 mg GAE/g DW) and total flavonoid (48.77 mg RE/100 g DW) concentrations were observed in samples dried at 80 °C, followed by those processed at 70 °C, indicating a temperature-dependent enhancement of these bioactive compounds. These results aligned with the findings of Turkmen et al. [43] who observed a gradual increase in the contents of total phenolic and flavonoid in cherry laurel fruits when heated from 50 °C to 70 °C. The high contents of phenolic and flavonoid compounds could be attributed to the release of bound compounds due to cell wall disruption and disruption of the esterified and glycosylated bond [44]. In addition, new Maillard-derived phenolic structures might form after drying [45]. This non-enzymatic browning process involves complex condensation and polymerization reactions between reducing sugars and amino acids, potentially generating additional phenolic compounds during thermal processing. The reaction generated various bioactive compounds, including melanoidins, aromatic compounds, heterocyclic compounds, and reducing ketones, which collectively contributed to the elevated phenolic and flavonoid contents [27,45].

The quality of oven-dried *S. grosvenorii* fruit was evaluated by quantifying ascorbic acid value, which is highly susceptible to degradation under abiotic stresses, including heat, light, and oxygen exposure [46]. As shown in Figure 4d, the ascorbic acid content of fresh fruit was 698.81 ± 2.71 mg/100 g DW, and this content dropped significantly after drying, with values ranging between 66.42 ± 3.41 mg/100 g DW and 170.50 ± 5.64 mg/100 g DW. Notably, the highest content of ascorbic acid was observed in samples dried at 70 °C, contrasting with research by Llavata et al. [47], who reported the maximal ascorbic acid preservation in apple pomace dried at 80 °C. The markedly lower ascorbic acid content in samples dried at 40 °C may be attributed to prolonged processing times, which facilitate oxidative degradation as well as concurrent non-enzymatic browning reactions such as caramelization and Maillard reaction [48]. The slow moisture diffusion at lower temperatures likely extended thermal exposure, thereby exacerbating ascorbic acid loss.

### 3.5. Antioxidant Activity

**Figure 5:**
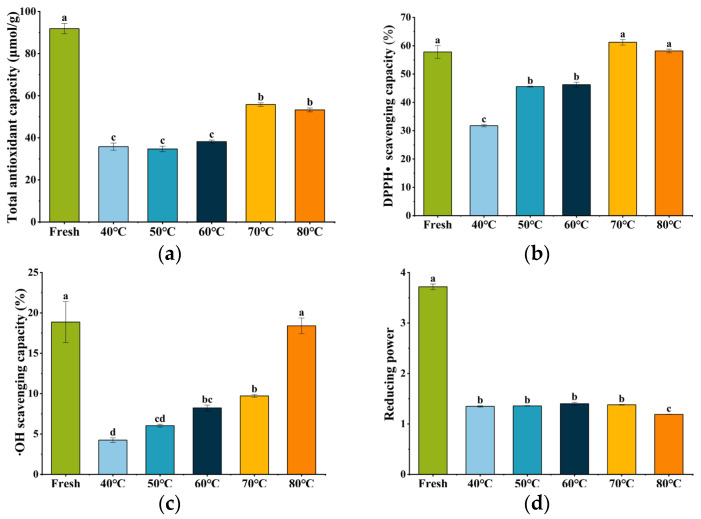
Antioxidant activity of dried *S. grosvenorii* at different hot-air temperatures. (**a**) Total antioxidant capacity; (**b**) DPPH• scavenging capacity; (**c**) •OH scavenging capacity; (**d**) reducing power. Different letters in the above columns indicate significant differences among drying temperature treatment groups (*p* < 0.05).

Evaluating antioxidant activity requires a multi-method approach due to the complex composition of plant materials and potential interactions between their bioactive constituents [29]. In this study, the antioxidant activity of *S. grosvenorii* fruit was comprehensively assessed using four assays: T-AOC, DPPH• and •OH radical scavenging capacities, and RP (Figure 5). Fresh fruit exhibited baseline values of 91.88 ± 2.47 μmol/g (T-AOC), 57.79 ± 2.26% (DPPH• scavenging capacity), 18.87 ± 2.54% (•OH scavenging capacity), and 3.71 ± 0.06 (RP). The dried samples exhibited lower antioxidant activity compared to the fresh fruit, which correlated with substantial reductions in both mogroside and ascorbic acid content. This observed decrease in antioxidant capacity can be attributed to the thermal degradation of key bioactive compounds in *S. grosvenorii* fruit during the drying process.

As presented in Figure 5a, the maximum T-AOC of 55.78 μmol/g was achieved at 70 °C, representing a 1.6-fold increase compared to the lowest T-AOC value observed at 50 °C. This substantial reduction at lower temperatures may result from prolonged drying durations, which promote the structural degradation of thermolabile compounds through delayed water removal and extended oxidative stress [49]. Consistent with the T-AOC results, samples dried at 70 °C also exhibited peak DPPH radical scavenging activity (61.20 ± 0.98%) (Figure 5b), correlating with their elevated phenolic, flavonoid, and ascorbic acid contents [48]. These findings aligned with a previous study by Wang et al. [15], who noted the optimal DPPH• scavenging capacity in bee pollen dried at 75 °C, suggesting that intermediate drying temperatures may generally favor antioxidant retention across different plant materials.

Both drying temperature and duration significantly influenced the •OH scavenging capacity (*p* < 0.05) (Figure 5c). A positive correlation was observed between scavenging activity and drying temperature, with the highest activity in samples dried at 80 °C (18.40 ± 0.97%), followed by those at 70 °C (9.72 ± 0.17%). Previous research has demonstrated that •OH scavenging capacity was strongly associated with phenolic and flavonoid contents [50]. These bioactive compounds could effectively neutralize •OH by transferring a hydrogen atom or a single electron, thereby scavenging hydroxyl radicals [51]. Notably, the trend in •OH scavenging ability observed in *S. grosvenorii* strongly correlated with measured TP and TF levels, confirming their synergistic role in radical mitigation. 

The RP value exhibited a parabolic relationship with drying temperature, initially increasing and then declining at higher temperatures. The maximum RP value (1.40 ± 0.03) was documented at 60 °C, with samples dried at 70 °C showing similar activity (1.38 ± 0.01) (Figure 5d). Wu et al. [52] reported that infrared freeze-drying at 60 °C yielded the highest RP of *Cordyceps militaris.* Rashid et al. [48] consistently found that infrared drying at 70 °C could improve the RP of sweet potatoes.

### 3.6. Multivariate Statistical Analysis

**Figure 6:**
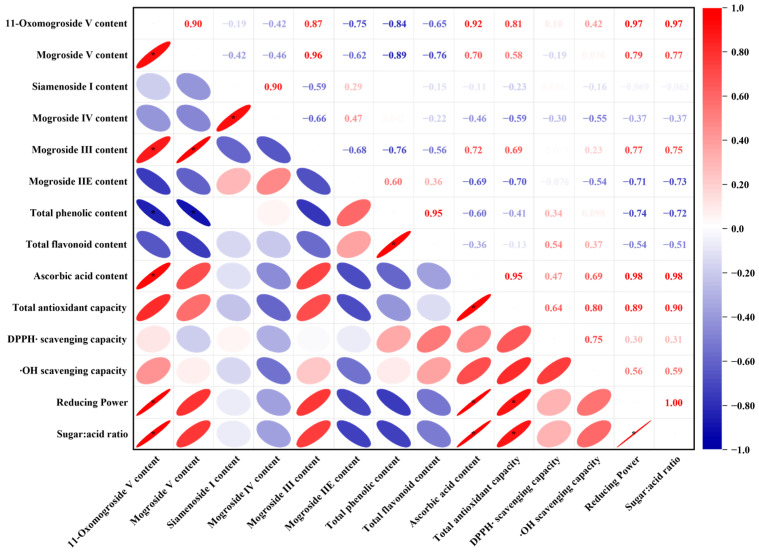
The correlations between the contents of mogrosides, total phenolic, total flavonoid, ascorbic acid, and antioxidant capacity parameters of *S. grosvenorii* fruit at different hot-air temperatures. * Means values with superscripts are significantly different, *p* < 0.05. Red is positive correlation, and blue is negative correlation (1 = positive correlation, 0 = no correlation, and −1 = negative correlation).

The correlations between the sugar/acid ratio, contents of mogrosides, total phenolic, total flavonoid, ascorbic acid, and antioxidant capacity parameters in dried *S. grosvenorii* fruit were analyzed (Figure 6). The result showed that the sugar/acid ratio was positively correlated with 11-O-MV content and T-AOC. The data also demonstrated that ascorbic acid served as a major determinant of antioxidant activity, exhibiting strong positive correlations with T-AOC and DPPH• and •OH scavenging capacity. Mogrosides, the principal bioactive constituents of *S. grosvenorii* fruit, exhibit significant anti-inflammatory, antioxidant, and hypoglycemic activities [6]. The results showed a positive correlation between specific mogrosides (MIII, MV, and 11-O-MV) and RP. Previous in vitro studies by Mo et al. [53] demonstrated that mogrosides significantly attenuated oxidative stress in H_2_O_2_-challenged mice, as evidenced by a marked reduction in MDA levels. This finding substantiates the antioxidant efficacy of mogrosides observed in our current study. Furthermore, MV content exhibited positive correlations with mogrosides 11-O-MV and MIII, and negative correlations with mogrosides SIA, MIV, and MIIE. These correlation patterns strongly suggest the presence of interconversion pathways among different mogrosides during the processing of *S. grosvenorii* fruit [9].

## 4. Conclusions

In the present study, the Midilli–Kucuk model provided the most accurate prediction of the drying characteristics of *S. grosvenorii* fruit. The L* color value and mogroside content decreased as the drying temperature increased. Although drying at 40 °C was beneficial for retaining MV content, the drying time (420 h) was extremely long and might be unrealistic for industrial practice. In contrast, samples dried at 70 °C exhibited superior performance in terms of functional constituents and antioxidant capacity. Importantly, the drying process increased phenolic and flavonoid content compared with fresh *S. grosvenorii* fruit. The drying temperature significantly affected antioxidant capacity, with an optimal temperature maintaining higher antioxidant capacity in *S. grosvenorii* fruit. Correlation analysis further indicated that mogrosides and ascorbic acid possessed potent antioxidant potential. These findings suggest that controlled hot-air drying could maintain acceptable bioactive composition and antioxidant potential, making *S. grosvenorii* suitable for the development of functional food ingredients and dietary supplements. Overall, this research provides experimental evidence for the selection and industrial application of drying methods for *S. grosvenorii* fruit, thereby offering a foundation for further improvements in the quality of dried *S. grosvenorii* fruit.

## Figures and Tables

**Table 1:** Mathematical models applied to drying curves.

Model Classification	Model Name	Equation
Semi-theoretical models	Lewis	*MR* = exp (−*kt*)
	Page	*MR* = exp (−*kt^n^*)
	Henderson–Pabis	*MR* = *a* exp (−*kt*)
	Verma	*MR* = *a* exp (−*kt*) + (1 − *a*) exp (−*gt*)
	Logarithmic	*MR* = *a* exp (−*kt*) + *c*
	Diffusion Approximation	*MR* = *a* exp (−*kt*) + (1 − *a*) exp (−*kbt*)
	Two-Term exponential	*MR* = *a* exp (−*k*_0_*t*) + *b* exp (−*k*_1_*t*)
	Midilli–Kucuk	*MR* = *a* exp (−*kt^n^*) + *bt*
Empirical models	Wang–Singh	*MR* = 1 + *at* + *bt*^2^
	Parabolic	*MR* = *a* + *bt* + *ct*^2^

Note: *MR*, moisture ratio (%); *k*, *n*, *a*, *g*, *c*, *b*, parameters of each applied model; *t*, drying time (h).

**Table 2:** Midilli–Kucuk model and Wang and Singh model parameters for *S. grosvenorii* fruit.

Model	Temperature (°C)	Model Parameters				*R* ^2^	*χ* ^2^	RMSE
		*a*	*b*	*k*	*n*			
Midilli–Kucuk	40	0.99281	−0.000949	0.00171	1.04388	0.9998	2.06 × 10^−5^	0.00454
	50	0.97879	−0.0003096	0.00310	1.25497	0.9991	0.11 × 10^−5^	0.01042
	60	0.99745	−0.0001205	0.01696	1.06625	0.9998	1.98 × 10^−5^	0.00445
	70	0.99999	−0.0005970	0.05375	0.85930	0.9998	2.11 × 10^−5^	0.00459
	80	0.99996	−0.0013200	0.09432	0.78167	0.9997	3.68 × 10^−5^	0.00606

Note: Parameters of each applied model (*a*, *b*, *k*, and *n*), coefficient of determination (*R*^2^), chi-square (*χ*^2^), and root-mean-square error (RMSE).

## Data Availability

The original contributions presented in this study are included in the article/Supplementary Materials. Further inquiries can be directed to the corresponding authors.

## Supplementary Materials

The following supporting information can be downloaded at https://www.mdpi.com/article/10.3390/foods15020335/s1, Table S1: Information on different model parameters to *S. grosvenorii* fruit.
