# Supplementary material for: Influence of Drying Temperature on Quality Characteristics and Drying Kinetics of Siraitia grosvenorii Fruit
Source: Foods. 2026 Jan 16;15(2):335. doi: 10.3390/foods15020335 (PMC12841080; doi:10.3390/foods15020335)
Supplement: Supplementary file 1 [file foods-15-00335-s001.zip › foods-4057961-supplementary.pdf]

Table S1: Information on different model parameters to *S. grosvenorii* fruit

Model classification	Model	Temperature/°C	Parameters	R^2	χ^2	RMSE
Semi-theoritical models	Lewis	40	k=0.0044	0.9443	5.00×10^{-3}	0.0707
		50	k=0.0118	0.9731	2.78×10^{-3}	0.0527
		60	k=0.0229	0.9968	2.97×10^{-4}	0.0172
		70	k=0.0357	0.9974	2.21×10^{-4}	0.0149
		80	k=0.0531	0.9983	1.55×10^{-4}	0.0125
	Page	40	k=0.0004, n=1.4266	0.9861	1.29×10^{-3}	0.0359
		50	k=0.0026, n=1.3292	0.9961	4.21×10^{-4}	0.0205
		60	k=0.0154, n=1.0991	0.9995	4.61×10^{-5}	0.0068
		70	k=0.0396, n=0.9704	0.9977	2.18×10^{-4}	0.0148
		80	k=0.0636, n=0.9432	0.9989	1.09×10^{-4}	0.0104
	Henderson and Pabis	40	a=1.0902, k=0.0049	0.9568	3.99×10^{-3}	0.0632
		50	a=1.0766, k=0.0127	0.9800	2.16×10^{-3}	0.0465
		60	a=1.0260, k=0.0235	0.9975	2.42×10^{-4}	0.0156
		70	a=0.9895, k=0.0353	0.9975	2.28×10^{-4}	0.0151
		80	a=0.9924, k=0.0528	0.9984	1.68×10^{-4}	0.0130
	Verma	40	a=6.1573, k=0.0009, g=0.0004	0.9998	2.13×10^{-5}	0.0046
		50	a=55.5269, k=0.0044, g=0.0043	0.9978	2.44×10^{-5}	0.0156
		60	a=38.4494, k=0.0150, g=0.0148	0.9998	2.01×10^{-5}	0.0045
		70	a=0.0445, k=1259.3593, g=0.0342	0.9980	2.03×10^{-5}	0.0143

Model classification	Model	Temperature/°C	Parameters	R^2	χ^2	RMSE
		80	a=0.0790, k=5.7869, g=0.0493	0.9992	9.08×10^{-5}	0.0095
	Logaritmic	40	a=2.5907, k=0.0012, c=−1.5949	0.9998	1.92×10^{-5}	0.0044
		50	a=1.2705, k=0.0077, c=−0.2513	0.9978	2.53×10^{-4}	0.0159
		60	a=1.0483, k=0.0208, c=−0.0406	0.9996	3.92×10^{-5}	0.0063
		70	a=0.9986, k=0.0338, c=−0.0150	0.9979	2.15×10^{-4}	0.0147
		80	a=0.9838, k=0.0544, c=0.0112	0.9985	1.77×10^{-4}	0.0133
	Diffusion Approximation	40	a=5.9939, b=0.4974, k=0.0009	0.9998	2.13×10^{-5}	0.0046
		50	a=44.7297, b=0.9757, k=0.0044	0.9978	2.44×10^{-4}	0.0156
		60	a=32.5985, b=0.9873, k=0.0150	0.9998	2.01×10^{-5}	0.0045
		70	a=0.0445, b=0.0362, k=0.9442	0.9980	1.85×10^{-4}	0.0136
		80	a=0.9875, b=0.0000, k=0.0548	0.9985	1.82×10^{-4}	0.0135
	Two-Term exponential	40	a=26.4699, b=−25.4745, k_0=0.0007, k_1=0.0006	0.9998	1.92×10^{-5}	0.0044
		50	a=44.6614, b=−43.6443, k_0=0.0048, k_1=0.0047	0.9982	2.19×10^{-4}	0.0148
		60	a=1.0650, b=−0.0650, k_0=0.0244, k_1=15.8819	0.9986	1.57×10^{-4}	0.0125
		70	a=0.9555, b=0.0445, k_0=0.0342, k_1=18.9813	0.9980	2.26×10^{-4}	0.0150
		80	a=0.9210, b=0.0790, k_0=0.0493, k_1=3.4572	0.9992	1.09×10^{-4}	0.0104
	Midilli-Kucuk	40	a=0.99281, b=−0.000949, k=0.00171, n=1.04388	0.9998	2.06×10^{-5}	0.00454
		50	a=0.97879, b=−0.0003096, k=0.00310, n=1.25497	0.9991	0.11×10^{-5}	0.01042
		60	a=0.99745, b=−0.0001205, k=0.01696, n=1.06625	0.9998	1.98×10^{-5}	0.00445

Model classification	Model	Temperature/°C	Parameters	R^2	χ^2	RMSE
		70	*a*=0.99999, *b*=−0.0005970, *k*=0.05375, *n*=0.85930	0.9998	2.11×10^{-5}	0.00459
		80	*a*=0.99996, *b*=−0.0013200, *k*=0.09432, *n*=0.78167	0.9997	3.68×10^{-5}	0.00606
Empirical models	Wang-Singh	40	*a*=−0.0030, *b*=0.0000	0.9998	2.15×10^{-5}	0.0046
		50	*a*=−0.0085, *b*=0.0000	0.9996	4.30×10^{-5}	0.0066
		60	*a*=−0.0163, *b*=0.0001	0.9902	9.57×10^{-4}	0.0309
		70	*a*=−0.0250, *b*=0.0002	0.9663	3.12×10^{-3}	0.0559
		80	*a*=−0.0421, *b*=0.0005	0.9886	1.16×10^{-3}	0.0341
	Parabolic	40	*a*=0.9941, *b*=−0.0030	0.9998	1.73×10^{-5}	0.0042
		50	*a*=1.0046, *b*=−0.0086	0.9996	4.18×10^{-5}	0.0065
		60	*a*=0.9468, *b*=−0.0148, *c*=0.0001	0.9944	5.80×10^{-4}	0.0241
		70	*a*=0.9115, *b*=−0.0215, *c*=0.0001	0.9796	2.08×10^{-3}	0.0456
		80	*a*=0.9712, *b*=−0.0400, *c*=0.0004	0.9899	1.20×10^{-3}	0.0347

Note: Parameters of each applied model (*a*, *b*, c, g, *k*, and *n*), coefficient of determination (R^2), chi-square (χ^2), and root-mean-square error (RMSE)
